# Risk-Aware Resource Allocation Strategy for Target Tracking in a Cognitive Radar Network

**DOI:** 10.3390/s26113299

**Published:** 2026-05-22

**Authors:** Ji Ye Lee, Jongho Park

**Affiliations:** 1 Department of Military Digital Convergence, Ajou University, Suwon 16499, Republic of Korea; jkllffdsa@ajou.ac.kr; 2 School of Mechanical Engineering, Kookmin University, Seoul 02707, Republic of Korea

**Keywords:** cognitive radar, resource allocation, target tracking, operational risk assessment, convex optimization, Second-Order Cone Programming (SOCP)

## Abstract

Cognitive radar has been developed to use feedback from its operating environment, obtained from a beam, to make resource allocation decisions by solving optimization problems. Previous works focused on target tracking accuracy by designing an evaluation metric for an optimization problem. However, in practical real-world scenarios, both the target tracking performance of cognitive radar and its operational perspective should be considered. In this study, the usage of an operational risk score in the allocation of radar resources is proposed for a cognitive radar framework. Resource allocation regarding radar dwell time is considered to reflect the operational significance of the target’s priority level. The dwell time allocation problem is solved through Second-Order Cone Programming (SOCP). Numerical simulations are performed to verify the effectiveness of the proposed framework. The results show that the proposed SOCP-based algorithm achieves comparable operational risk estimation performance to conventional methods while using fewer time resources, thereby improving overall resource efficiency in resource-constrained environments.

## 1. Introduction

A radar network is a system in which multiple radars work together and communicate with each other using information collected from the target [1,2,3]. Recently, based on the development of communication technology, radar networks have gained growing attention. Since tracking data can be utilized in a coordinated way, there are some benefits of radar networks compared to a conventional single-radar operation, such as an increased coverage area and increased accuracy of target tracking and detection [4,5]. For example, the integrated information from the radar network can be used to detect Low-Probability-of-Intercept (LPI) targets in a surveillance system, and recent studies have addressed resource management in networked surveillance scenarios under strict constraints [6]. Furthermore, research on improving maneuvering target tracking performance using state-of-the-art radar systems is actively being conducted [7].

In radar networks, research on resource management is an essential issue since the information shared through channels is affected by the allocated resources of each node. That is, the accuracy of the obtained information may vary depending on how the limited resources are used, even with the same geographical arrangement of the radar nodes [8,9,10,11,12]. Liu et al. [13] aimed to increase target state estimation accuracy and lower the false alarm rate of tracking a target in a cluttered environment. To solve this problem, a Collaborative Detection and Power Allocation (CDPA) scheme was developed, and the optimization problem was solved by using the Cramer–Rao Lower Bound (CRLB) as the analytical cost. On the other hand, Yan et al. [14] derived an optimization problem for multiradar–multitarget situations by considering which target to allocate to each radar and how to allocate the limited time resources of each radar to the target. Recently, to address more complex scenarios, joint resource allocation methods have been explored for distributed MIMO radar systems [15]. Furthermore, resource management has been extended to electronic warfare environments, such as enhancing stealth performance via counter-sorting approaches [16] and developing robust power allocation under severe external electromagnetic interference [17]. In addition to traditional tracking, adaptive scheduling algorithms have been proposed for 3-D target imaging [18], and recent trends have even expanded to integrated sensing and communications (ISAC) [19]. Recently, data-driven approaches, such as deep reinforcement learning, have also been investigated for adaptive resource distribution in cognitive radar networks [20]. Accordingly, the importance of online resource and mission scheduling tailored to dynamically changing operating conditions is also increasingly emphasized [21].

In some areas of radar-network research, an operational risk-aware approach is proposed to calculate operational risks using target state parameters and use this information for scheduling. Such an operational risk-based resource management technique has the advantage of an operational concept that can be efficiently applied to a scheduling algorithm [22,23,24,25,26,27,28]. While some recent studies explore radar operational risk environments from the perspective of an infiltrating target, such as UAV swarms avoiding detection [29], this study focuses on the system operator’s perspective. For the case of phased-array surveillance radar, Zhang et al. [30] designed an appropriate operational risk model of the target so that the prior information of the target could be fully utilized. Additionally, by designing a 2D dynamic priority table, the operational risk level of the target and the deadline of the task were considered comprehensively to calculate the priority. Furthermore, Li et al. [31] introduced a target prioritization algorithm based on three-way decision theory. Pang and Shan [32,33] calculated the target missing operational risk and the sensor radiation interception operational risk in a target tracking and detecting scenario. Once the operational risk was specified, resource management was conducted with the goal of minimizing the defined operational risk. To combat such operational risks, various optimization techniques have recently been investigated for the precise tracking of high-risk targets, such as high-speed maneuvering targets [34]. Unlike the aforementioned works focused on reducing operational risk, Katsilieris et al. [35] proposed a method to increase the operational risk accuracy of the target, where the operator was defined according to the operational purpose.

To learn the environment and plan the next action in a human-like way, cognitive control systems have recently been actively studied [36,37,38,39,40,41]. In [42], a perception–action cycle, which is a distinguishing feature of a cognitive dynamic system, was mathematically modeled. This cognitive control framework was later applied to radar systems to establish the concept of cognitive radar. In [43], the Predicted Conditional Cramer–Rao Lower Bound (PC-CRLB), an appropriate evaluation metric for predicting the tracking performance in a cognitive radar environment, was developed, and the optimization problem was solved using an SOCP. However, these recent cognitive radar studies only considered the target state among the full set of features of the environment. In reality, both the target state and the operational risk, such as the safety of assets, should be considered, especially in the case of air defense radar. In a battlefield situation, accurate assessment of the operational risk of the target is a key requirement for operators.

From this point of view, we specifically develop two key elements in this study. We propose a general condition that can accommodate the operator’s requirements, and we mathematically verify an example case of the condition. In other words, a cognitive radar resource management model that includes the operational risk of the target is presented. The proposed model is better aligned with practical operational requirements, and operational flexibility is also increased. In addition, a new objective function is proposed to better express the operational risk-based performance. Subsequently, the performance is verified using numerical simulation.

The major contributions of this paper are summarized as follows:

- First, we formulate the operational risk estimation problem as a state estimation problem to utilize the convex properties of Second-Order Cone Programming (SOCP).
- Second, we propose an SOCP-based resource allocation algorithm that yields a computationally efficient globally optimal solution for the formulated PC-CRLB-based surrogate optimization problem.
- Finally, simulation results demonstrate that the proposed method improves resource efficiency over conventional heuristics while maintaining comparable operational risk estimation accuracy.

A detailed comparison of these recent resource management approaches in radar networks is summarized in Table 1.

This paper is organized as follows. In Section 2, the necessity of an operational risk-aware approach in radar systems is discussed. Then, an operational risk-based approach framework for cognitive radar is presented in Section 3. In Section 4, a dwell-time allocation example is given, and it is validated via numerical simulation in Section 5. Finally, the conclusion is given in Section 6.

## 2. The Necessity of an Operational Risk-Aware Approach in Radar Systems

According to [43], an object that poses an operational risk to a valuable asset can be defined as a hazard, and the potential loss due to this hazard is defined as an operational risk. In a safety-critical environment, a flying enemy object can be considered a potential operational risk. Risks can be labeled with numbers or letters in consideration of kinetic information as well as other features such as target type and intent. This series of processes is called operational risk assessment. Contrary to operational risk, which is a predicted loss after an event occurs, this labeled value represents the current relationship between the observed target and the asset. Conceptually, the operational risk has the same meaning as the operational risk label [35], and it is defined as a normalized value between 0 and 1. However, operationally or analytically, a monotonic unnormalized surrogate score may also be used to facilitate convex optimization, as will be detailed in later sections.

**Figure 1:**
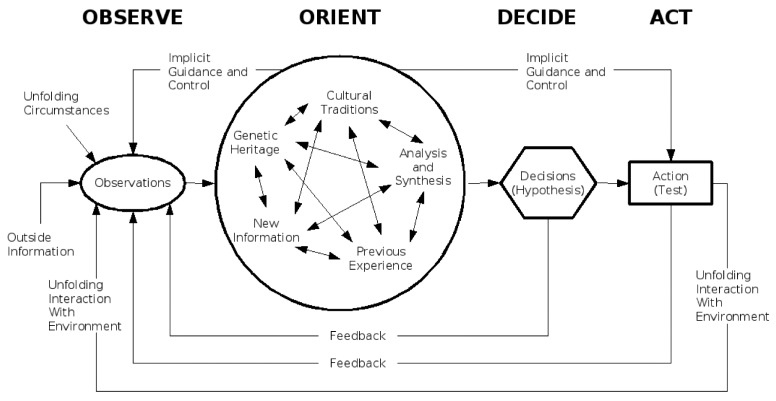
Observe–Orient–Decide–Act (OODA) loop cycle used to make decisions [27].

The Observe–Orient–Decide–Act (OODA) loop shown in Figure 1 is a well-known decision-making process in the time-critical surveillance domain, and this method can be applied in a safety-critical monitoring environment [27]. An observed raw measurement should be assessed so it can be used to make tactical decisions, and operational risk assessment can be seen as an example of such a process. In the real world, operational risk assessment is performed by a human observing a target. However, if the operational risk can be determined by processing only kinetic information, the probability distribution of the operational risk can be mathematically calculated using the probability distribution of the kinetic information [35]. Then, the operator can decide whether to respond to the detected target by considering the obtained operational risk label value and the resources of the currently available response resources. Since the assessed operational risk label value affects the determination of engagement, the accuracy of the obtained operational risk label has operational importance.

## 3. Risk-Aware Approach Framework in Cognitive Radar Networks

### 3.1. System Model

**Figure 2:**
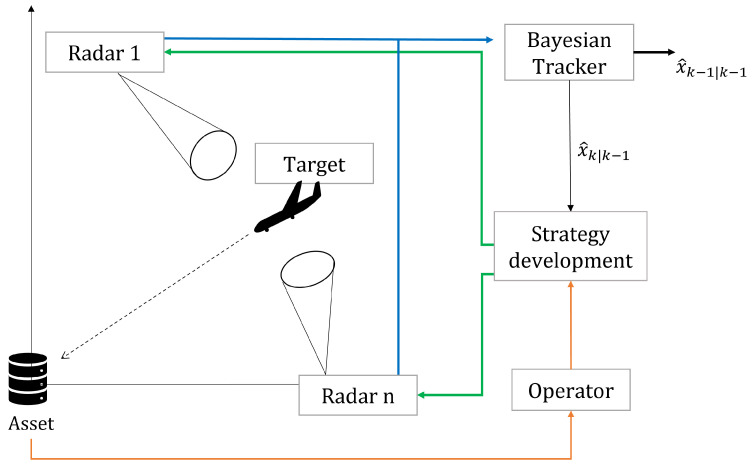
Overview of the proposed sensing strategy.

Figure 2 presents a representative surveillance scenario that is considered in this study.

The system model has the following assumptions.

1. N geographically distributed stationary monostatic radars are deployed in a two-dimensional plane.
2. Each radar has the same peak transmit power, which is fixed.
3. A single moving target exists.
4. The target is assumed to be a point target. For practical extended targets (e.g., aircraft represented as point clouds), standard clustering and Constant False Alarm Rate (CFAR) thresholding algorithms are assumed to be pre-applied at the signal processing level to extract the kinematic centroid for tracking.

The position of the *i*-th radar, $p_{i}$, and the target, $p_{k}$, at time $t_{k}$ can be denoted as follows:

(1) $$p_{i}={\left[x_{i}y_{i}\right]}^{T}$$

(2) $$p_{k}={\left[x_{k}y_{k}\right]}^{T}$$

The target state-transition model is given by:

(3) $$X_{k}=f\left(X_{k-1}\right)+V_{k-1}$$

where the target state $X_{k}={\left[x_{k}\;{\dot{x}}_{k}\;y_{k}\;{\dot{y}}_{k}\right]}^{T}$ denotes the position and velocity of the target and f(·) is a model function. $V_{k-1}$ is process noise, which has a Gaussian distribution with zero mean and covariance $Q_{k-1}$. Note that the target measurement of the *i*-th radar at time $t_{k}$ can be described using (1) and (2).

(4) $$\begin{array}{cc} z_{i,k} & ={\left[R_{i,k}\;\varphi_{i,k}\right]}^{T}+w_{i,k}\left(T_{i,k}^{d}\right)\\ \; & ={\left[∥p_{k}-p_{i}∥\;\mathrm{atan}2\left(y_{k}-y_{i},x_{k}-x_{i}\right)\right]}^{T}+w_{i,k}\left(T_{i,k}^{d}\right) \end{array}$$

where ∥·∥ is the Euclidean norm of the vector, $R_{i,k}$ is the range between the radar and the target, $\varphi_{i,k}$ is the bearing angle between the radar and the target, and $T_{i,k}^{d}$ is the dwell time of radar *i* at $t_{k}$. The measurement noise $w_{i,k}$ can be defined as follows:

(5) $$w_{i,k}\left(T_{i,k}^{d}\right)∼N\left(0,\;\Gamma_{i,k}\right)$$

(6) $$\Gamma_{i,k}=diag\left\{{\left(\sigma_{i,k}^{R}\right)}^{2},{\left(\sigma_{i,k}^{\varphi }\right)}^{2}\right\}$$

$N(0,\Gamma_{i,k})$ is the normal distribution, which has zero mean and covariance $\Gamma_{i,k}$, and diag{A} denotes a diagonal matrix with A as the value of the nonzero cells. In (6), $\sigma_{i,k}^{R}$ and $\sigma_{i,k}^{\varphi }$ are the theoretical measurement accuracy of the *i*-th radar, which can be represented by the following equations [1]:

(7) $$\sigma_{i,k}^{R}=\frac{c}{2\beta_{i}\sqrt{{SNR}_{i,k}}}\;\propto {\left(\beta_{i}\sqrt{\frac{T_{i,k}^{d}{\bar{\sigma }}_{i}}{R_{i,k}^{4}}}\right)}^{-1}$$

(8) $$\sigma_{i,k}^{\varphi }=\frac{0.628\;\theta_{i}^{3dB}}{\sqrt{{SNR}_{i,k}}}\;\propto {\left(\frac{\sqrt{\frac{T_{i,k}^{d}\;\bar{\sigma_{i}}}{R_{i,k}^{4}}}}{\theta_{i}^{3dB}}\right)}^{-1}$$

where c is the speed of light, $\beta_{i}$ is the effective bandwidth of the signal transmitted by the *i*-th radar, $\theta_{i}^{3dB}$ is the 3 dB beam width of the *i*-th radar antenna pattern, $\bar{\sigma_{i}}$ is the average target Radar Cross-Section (RCS), and $SNR_{i,k}$ is the signal-to-noise ratio, which is proportional to $T_{i,k}^{d}\bar{\sigma_{i}}/R_{i,k}^{4}$.

Using (1)–(8), the measurement equation can be given as

(9) $$Z_{k}=h\left(X_{k}\right)+W_{k}\left(u_{k}\right)$$

where $Z_{k}={\left[z_{1,k}^{T}\cdots z_{N,k}^{T}\right]}^{T}$ is the set of measurements of the radar network, $u_{k}={\left[T_{1,k}^{d}\cdots T_{N,k}^{d}\right]}^{T}$ is the radar-target dwell time allocation at time $t_{k}$, and $W_{k}\left(u_{k}\right)={\left[w_{1,k}^{T}\left(T_{1,k}^{d}\right)\cdots ,w_{N,k}^{T}\left(T_{N,k}^{d}\right)\right]}^{T}$ is the measurement noise, which has a Gaussian distribution with zero mean and covariance $R_{u_{k}}=blkdiag\left\{\Gamma_{1,k},\cdots ,\Gamma_{N,k}\right\}$.

### 3.2. Operational Risk Model

The operational risk function Φ(∗) considering operational requirements can be constructed as follows:

(10) $$\theta_{k}=\Phi \left(X_{k},a_{k}\right)$$

where $\theta_{k}$ is the operational risk of the target at time $t_{k}$ and $a_{k}$ is a variable vector that describes the surrounding operating environment (e.g., positions of assets, weights between kinetic parameters).

**Figure 3:**
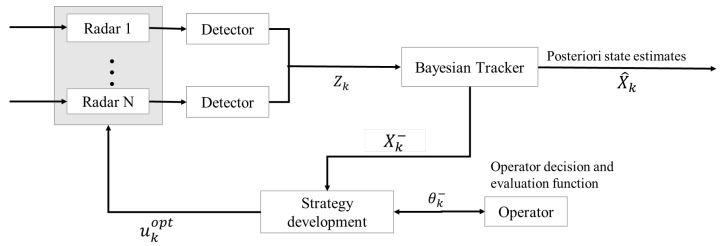
Cognitive action cycle of a radar network.

To update the state of the target by learning the surrounding environment, the radar network can be modeled as a cognitive action cycle, as shown in Figure 3.

According to the cognitive action cycle, high performance can be expected for the next measurement by pre-calculating the optimal solution of the predicted measurement. $Z_{k}^{↑}$, the predicted information for $Z_{k}$ considering the previous knowledge before $t_{k}$, can be defined as

(11) $$Z_{k}^{↑}≜h\left(X_{k}^{-}\right)+W_{k}\left(u_{k}\right),u_{k}\in U$$

where *U* is the candidate strategy set. $X_{k}^{-}∼N\left({\hat{X}}_{k|k-1},P_{k|k-1}\right)$ is the predicted state of $X_{k}$, where ${\hat{X}}_{k|k-1}$ and $P_{k|k-1}$ are the estimated state and covariance of the target at $t_{k-1}$ [2]. Inspired by the Bayesian rule in (12), the virtual state update $X_{k}^{-↑}$ can be expressed using $p(X_{k}^{-}|Z_{k}^{↑})$ [13]. 

(12) $$p\left(X_{k}^{-↑}|Z_{k}^{↑}\right)\propto p\left(X_{k}^{-},Z_{k}^{↑}\right)=p\left(Z_{k}^{↑}|X_{k}^{-}\right)p\left(X_{k}^{-}\right)$$

Since estimating $X_{k}^{-↑}$ is a Bayesian state estimation problem, its analytical solution is difficult to obtain. Instead, the estimated theoretical bound of PC-CRLB from [13] was utilized in this paper. Subsequently, the virtual operational risk $\theta_{k}^{-↑}$ can also be derived using $X_{k}^{-↑}$ and (10).

(13) $$\theta_{k}^{-↑}=\Phi \left(X_{k}^{-↑},a_{k}\right)$$

Since $\theta_{k}^{-↑}$ is the prediction of $\theta_{k}^{-}=\Phi \left(X_{k}^{-},a_{k}\right)$, its uncertainty $\Sigma_{\theta_{k}^{-}}^{↑}$ can be represented using the definition of the predicted conditional mean square error (PC-MSE) [43].

(14) $$\Sigma_{\theta_{k}^{-}}^{↑}=E_{X_{k}^{-},Z_{k}^{↑}}\left[{\left(\theta_{k}^{-}-\theta_{k}^{-↑}\right)}^{2}\right]$$

where $E_{x}\left[*\right]$ is expectation with respect to x.

It is worth noting that leaving the operational risk level as a simple empirical score makes it mathematically intractable for rigorous optimization. By converting the operational risk evaluation into a state estimation problem, we can utilize established mathematical metrics, such as the error covariance of the Kalman filter. This crucial transformation allows the formulation of a convex objective function, laying the foundation for finding a global optimum in resource allocation rather than relying on heuristic approximations.

Regarding the nature of the operational risk evaluation, while the underlying operational risk function model itself relies on a static, rule-based structure (e.g., operational risk increases as distance decreases), the calculated operational risk level is intrinsically dynamic. Since the operational risk value is strictly coupled with the target’s state $X_{k}$, it is dynamically updated at every time step *k* based on the real-time state predictions. This ensures that the resource allocation mechanism adaptively responds to the rapidly changing surrounding operating environment.

### 3.3. Constraint on the Risk Function

**Figure 4:**
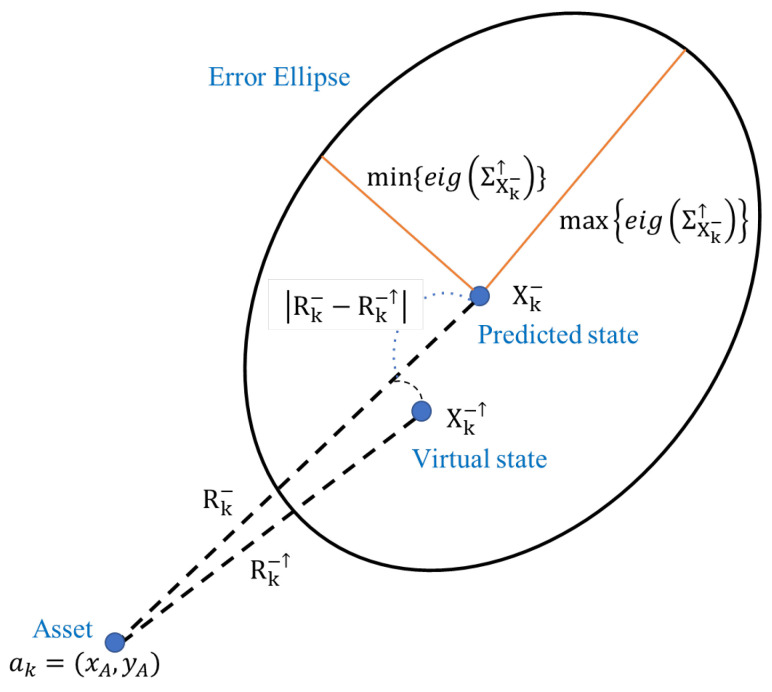
Asset protection scenario.

As a surveillance system, radar must provide the operator with good operational risk accuracy so that the operator can properly recognize the battlefield situation and respond accordingly. Since the estimation of $\theta_{k}^{-↑}$ is a Bayesian estimation problem [3], the efficiency can be expressed using the variance of the estimator expressed in (14). The method of calculating the variance may be different depending on the definition of $\Phi (X_{k},a_{k})$ in (10) with respect to the considered environment. In this study, operational risk function restrictions are derived considering a scenario in which the radar system protects critical infrastructure (e.g., high-value fixed facilities) from approaching targets, as shown in Figure 4.

The distance *R* between the asset and the target can be defined using the given asset position $a_{k}=\left(x_{A},y_{A}\right)$, where $x_{A}$ is the x-position and $y_{A}$ is the y-position of the asset, and the target state $X_{k}$ is as follows:

(15) $$a_{k}=\left(x_{A},y_{A}\right)$$

(16) $$R\left(a_{k},X_{k}\right)=\sqrt{{\left(x_{A}-x_{k}\right)}^{2}+{\left(y_{A}-y_{k}\right)}^{2}}$$

The distance between the target and the asset can be considered a parameter for calculating the operational risk. Therefore, $\theta_{k}$ can be expressed using (16), and *g* is the mapping used to compute the operational risk θ using (16):

(17) $$\Phi \left(X_{k},a_{k}\right)=g\left(R\left(a_{k},X_{k}\right)\right)=\theta_{k}$$

Because R is the distance, g(x) is defined only when x≥0, and we define the operational risk as a positive value.

In general, the operational risk function g(R) is required to be positive and monotonically decreasing with respect to the asset–target distance *R*, because a closer target should never be assigned a lower operational risk level than a farther one under the same operational context. This monotonicity ensures that any reduction in the distance error directly translates into an improvement in the operational risk estimation accuracy, regardless of the specific functional shape of g(·). In the remainder of this paper, we therefore focus on a practically important subclass of such functions, namely linear distance-based operational risk models, for which the relationship between the operational risk error and the position error can be characterized explicitly and used to design a convex optimization criterion.

Since the asset position is fixed, the range function R(∗) with respect to the target position is 1-Lipschitz continuous. Therefore, the distance error is bounded by the Euclidean norm of the position error:

(18) $$|R_{k}^{-}-R_{k}^{-↑}|\leq ∥p_{k}^{-}-p_{k}^{-↑}∥$$

where $p_{k}$ denotes the 2D position component $\left(x_{k},y_{k}\right)$ of the target state. Consequently, the problem of minimizing the operational risk estimation uncertainty can be directly linked to minimizing the spatial position uncertainty of the target.

### 3.4. Optimization Formulation and Dwell Time Allocation

Since the target state estimation problem is a Bayesian estimation problem, the efficiency can be expressed using its covariance. The PC-CRLB supplemented with the Bayesian Cramer–Rao Lower Bound (BCRLB) is a method commonly used to calculate the lower bound of covariance, and it is utilized in this study for performance evaluation in cognitive radar networks. In a situation where dwell time is a radar resource, the cost function $C(u_{k})$ of the resource allocation output $u_{k}$ can be expressed as $C\left(u_{k}\right)=1^{T}u_{k}$. If the constraint of the optimization problem is formulated to keep the operational risk accuracy from reaching a certain lower limit *P*, it can be expressed as follows.

(19) $$\begin{array}{c} \;\;\;\;\;\;\;\;\;\;\;\;\;\arg\min_{u_{k}}C\left(u_{k}\right)\\ \mathrm{subject}\;\mathrm{to}\;0\leq u_{k}\leq T_{k}^{d,max}\\ \;\;\;\;\;\;\;\;\;\;\;\;\;\;\;\;\;\;\;\;\;\;M(\theta_{k}^{-↑};u_{k})<P \end{array}$$

where $M(\theta_{k}^{-↑};u_{k})$ is a function that evaluates $\theta_{k}^{-↑}$. M can be defined using known performance measures of the covariance matrix. The optimal allocation strategy, $u_{k}^{opt}$, can be achieved by solving optimization problem (19). $u_{k}^{opt}$ can be applied to a radar network by controlling the measurement noise in (4).

The computational complexity of the SOCP is generally bounded by $O(M^{3})$, where *M* is the number of variables (i.e., the number of radars). Given the small number of radars in typical networked scenarios, this polynomial-time complexity ensures that the proposed resource allocation can be executed efficiently, satisfying the real-time operational constraints of the cognitive radar network.

## 4. An Example of the Operational Risk-Aware Approach

As a representative case that satisfies the aforementioned monotonic decreasing property, let us assume that the operational risk function g(·) takes the following linear form:

(20) $$g\left(R_{k}\right)=aR_{k}+b$$

where a<0, b>0, and $R_{k}$ is $R(a_{k},X_{k})$ in (16). In practical military applications, the parameters of the operational risk function (*a* and *b*) can be adaptively adjusted by human operators. For instance, an operator can assign a steeper slope (larger |a|) to a highly critical asset (e.g., an operational command post) to enforce stricter tracking accuracy compared to a less critical target, thereby providing operational flexibility.

For the linear operational risk function in (20), the operational risk estimation error variance satisfies the following bounds utilizing the 1-Lipschitz property from (18):

(21) $$\begin{array}{cc} E_{X_{k}^{-},Z_{k}^{↑}}\left[{\left(\theta_{k}^{-}-\theta_{k}^{-↑}\right)}^{2}\right] & =a^{2}E_{X_{k}^{-},Z_{k}^{↑}}\left[{\left(R_{k}^{-}-R_{k}^{-↑}\right)}^{2}\right]\\ \; & \leq a^{2}E_{X_{k}^{-},Z_{k}^{↑}}[∥p_{k}^{-}-p_{k}^{-↑}{∥}^{2}]\\ \; & =a^{2}\mathrm{tr}\left(\Sigma_{p,k}^{↑}\right)\\ \; & \leq 2a^{2}\lambda_{\max}\left(\Sigma_{p,k}^{↑}\right) \end{array}$$

where $\Sigma_{p,k}^{↑}$ is the positional submatrix of the predicted conditional covariance matrix, tr(∗) denotes the trace operation, and $\lambda_{\max}\left(*\right)$ is the maximum eigenvalue.

Because the true error covariance $\Sigma_{p,k}^{↑}$ is generally analytically intractable in nonlinear filtering, we adopt the positional submatrix of the PC-CRLB, denoted as $B_{p,k}^{-↑}$, as a tractable lower-bound surrogate of the position uncertainty. The eigenvalues of this covariance surrogate matrix determine the principal axes of the error ellipse; specifically, the squared semi-major axis length is proportional to the maximum eigenvalue $\lambda_{\max}\left(B_{p,k}^{-↑}\right)$. The detailed derivation of this surrogate is provided in Appendix A.

Therefore, minimizing $\lambda_{\max}\left(B_{p,k}^{-↑}\right)$ serves as a highly effective design objective for implicitly bounding and improving the operational risk estimation accuracy. Consequently, $M(\theta_{k}^{-↑};u_{k})$ in (19) is defined as  

(22) $$M\left(\theta_{k}^{-↑};u_{k}\right)=\lambda_{\max}\left(B_{p,k}^{-↑}\right).$$

Then, (19) can be transformed into an SOCP [43].

The rationale for transforming and solving this problem via Second-Order Cone Programming (SOCP) is based on the trade-off between expressive power and computational efficiency. Linear Programming (LP) is too simple to handle the nonlinear geometric properties of the error covariance matrix. Conversely, Semidefinite Programming (SDP) offers high expressiveness for covariance matrices but incurs excessive computational burden, making it unsuitable for real-time radar scheduling. SOCP provides a practical mathematical compromise: it strictly guarantees a global optimum through its convexity while maintaining low computational complexity that is highly feasible for real-time cognitive radar operations.

**Figure 5:**
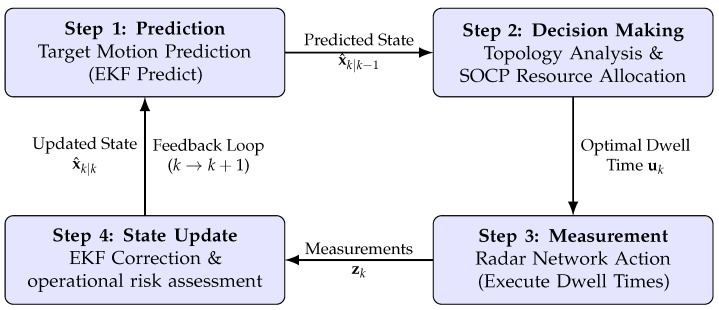
System flowchart of the proposed operational risk-based cognitive radar resource allocation framework.

Algorithm 1 explains the full flow of the system, where $k_{max}$ is the index of the final time step, *N* is the number of radars, and the Extended Kalman Filter (EKF) is used as a Bayesian tracker. The corresponding block diagram of this workflow is presented in Figure 5.
**Algorithm** **1** Target tracking process in the cognitive radar framework  1:**Initialization:**  2:$t_{k}\leftarrow 1$  3:${\hat{X}}_{0|0}\leftarrow X_{0}$  4:$P_{0|0}\leftarrow P_{0}$  5:**while** $t_{k}<t_{k_{\max}}$ **do**                                                                                            ▷**Time loop**  6:      **Step 1. Estimation**  7:      ${\hat{X}}_{k|k-1}\leftarrow f\left({\hat{X}}_{k-1|k-1}\right)$  8:      $A_{k}\leftarrow$ Jacobian of *f* at $t_{k}$  9:      $P_{k|k-1}\leftarrow A_{k}P_{k-1|k-1}A_{k}^{T}+Q_{k-1}$10:        **Step 2. Decision making**11:       Construct the positional PC-CRLB surrogate $B_{p,k}^{-↑}$ from the predicted state12:       Calculate $\lambda_{\max}\left(B_{p,k}^{-↑}\right)$13:       Formulate the optimization problem in (19)14:       Obtain $u_{k}$ by solving the SOCP15:       **Step 3. State update**16:       ${\hat{X}}_{k|k}\leftarrow {\hat{X}}_{k|k-1}$17:       **for** i=1 to *N* **do**18:             **if** $u_{i,k}>0$ **then**19:                  Acquire $z_{i,k}=h_{i}\left(X_{k}\right)+w_{i,k}\left(T_{i,k}^{d}\right)$20:                  Compute the Jacobian $H_{i,k}$ at ${\hat{X}}_{k|k-1}$21:                  Calculate the Kalman gain22:                  Update ${\hat{X}}_{k|k}$ and $P_{k|k}$23:             **end if**24:        **end for**25:        $t_{k}\leftarrow t_{k}+1$26:**end while**

## 5. Simulation Results

**Figure 6:**
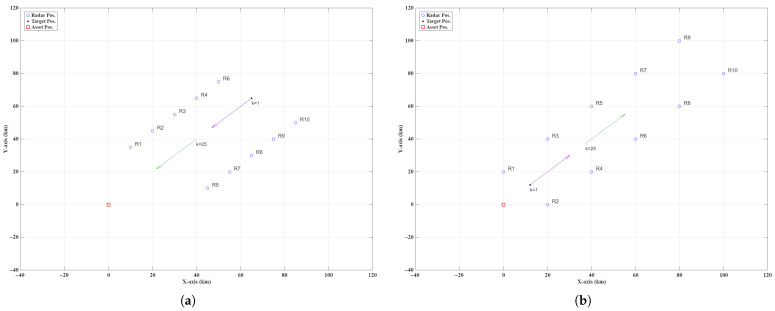
Two scenarios of radar–target–asset deployment. (**a**) Scenario 1: Gathered radars approaching assets. (**b**) Scenario 2: Scattered radars moving away from assets.

A numerical simulation was conducted to assess the performance of the proposed algorithm. A system of 10 monostatic vehicle-mounted radars was considered. Since each radar was mounted on a vehicle, there was a limited dwell time, and efficient resource allocation was important in maximizing the capacity of the radar. Simulations were conducted on two different scenarios to demonstrate the robustness of the proposed algorithm (see Figure 6) For consistency across the scenarios, the radars are sequentially numbered from R1 to R10 in ascending order of their X-axis coordinates.

We assumed all radars to have the same invariant effective bandwidth and average RCS: $\beta_{i}$ = 1/3 MHz, $\theta_{i}^{3dB}$ = 10 mrad, and ${\bar{\sigma }}_{i|k:1:k-1}$ = 1 m^2^. The sampling interval was set to 10 s to simulate a long-range surveillance scenario, and the simulation was conducted over 50 time frames. The target was tracked using a nearly constant-velocity model of the EKF [43]. $Q=\left[\begin{array}{cc} ▵T^{3}/3 & ▵T^{2}/2\\ ▵T^{2}/2 & ▵T \end{array}\right]⊗\kappa I_{2}$, where $I_{2}$ is the 2×2 identity matrix, ⊗ is the Kronecker product, κ is $1\;m^{2}/s^{3}$, and $P_{0}^{-}=250^{2}diag\left\{1,1,2/▵T^{2},2/▵T^{2}\right\}m^{2}$. Local targets from multiple radars were associated as a single system state using the sequential measurement update method of the Extended Kalman Filter [3]. The radars were considered stationary and the target moved at constant speed. While the conceptual operational risk label can be normalized to the range [0,1], the numerical simulation uses a linear unnormalized surrogate operational risk score, $\theta_{k}=-R_{k}+80,000$, where $R_{k}$ denotes the asset–target distance in meters. This surrogate preserves the monotonic relationship between distance and operational risk, while making the resulting operational risk error directly proportional to the underlying distance error. Therefore, the reported operational risk-related metric in the simulation should be interpreted as a scaled operational risk error rather than a normalized operational risk label. Furthermore, to transparently demonstrate the performance limits of the proposed SOCP-based resource allocation algorithm, we assumed an ideal detection environment without missed detections ($P_{d}=1$) and false alarms ($P_{fa}=0$). Incorporating probabilistic detection and clutter would require complex data association logic, which could obscure the pure efficacy of the Risk-Aware Resource Allocation Strategy. Detailed parameters used in the simulation are listed in Table 2.

**Figure 7:**
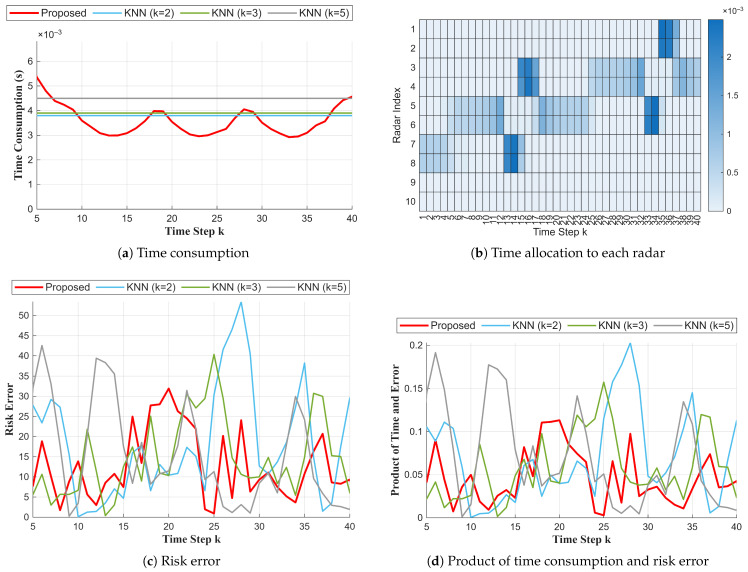
Simulation results of Scenario 1 (gathered radars approaching assets). (**a**) Total time resource consumption per frame. (**b**) Dwell time allocation distribution across the radar network. (**c**) Risk error representing the estimation accuracy of the operational risk. (**d**) Product of time consumption and Risk error, indicating the overall resource efficiency.

**Figure 8:**
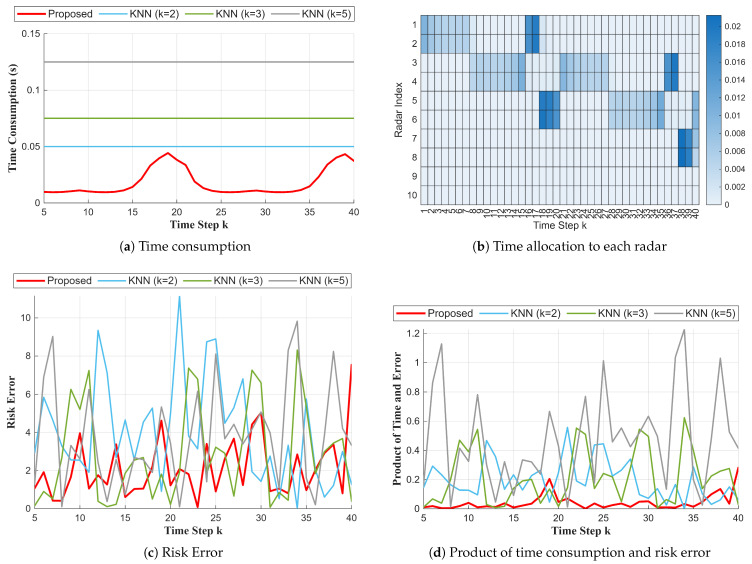
Simulation results of Scenario 2 (scattered radars moving away from assets). (**a**) Total time resource consumption per frame. (**b**) Dwell time allocation distribution across the radar network. (**c**) Risk error representing the estimation accuracy of the operational risk. (**d**) Product of time consumption and risk error, indicating the overall resource efficiency.

The results are shown in Figure 7 and Figure 8. The proposed algorithm was compared with the KNN algorithm, a representative distance-based heuristic approach, which allocates limited time resources to the nearest *k* radars. Regarding the selection of the parameter *k* in the KNN algorithm, determining an absolute ’optimal *k*’ for a dynamically moving target is inherently impractical in real-world operations. Therefore, in our experiments, we evaluated three representative values (k=2,3, and 5) to provide a comprehensive comparison. Specifically, k=2 represents the theoretical minimum required for 2D position estimation, highlighting the vulnerability of distance-based methods to poor geometric topology (GDOP). k=3 serves as the standard baseline widely used in the multistatic radar tracking literature. Finally, k=5 represents a resource-heavy scenario (utilizing 50% of the network) to demonstrate the inefficiency of purely adding more sensors. To ensure a fair and rigorous comparison, the KNN algorithm was configured to allocate a constant, fixed dwell time to the selected *k* radars at every time step. This fixed time resource was empirically tuned for each *k* setting prior to the evaluation so that the overall average risk error of the KNN algorithm exactly matched that of the proposed algorithm. By establishing this equal-accuracy baseline, the pure resource efficiency of the algorithms can be directly and objectively compared. Note that the risk error and the product metric graphs are plotted starting from $t_{k}=5$ to exclude the initial transient phase. During the initial time steps, the Extended Kalman Filter (EKF) experiences large estimation errors before the filter converges. Omitting this transient phase allows for a clearer and more accurate comparison of the steady-state tracking performance.

Figure 7a shows the time consumption comparison between the proposed algorithm and the KNN algorithms in Scenario 1. As designed, the KNN algorithms consume a constant amount of time resources at all frames to maintain the target tracking accuracy. In contrast, the proposed algorithm generally requires less time than the KNN baselines through adaptive, wave-like dwell-time allocation. It efficiently adjusts its resource allocation based on the evolving geometric topology, seamlessly increasing resources during critical topological transitions and conserving them elsewhere. For more details on how these resources are distributed, Figure 7b shows a heatmap of the proposed algorithm. It indicates that the proposed algorithm flexibly adapts to changes in the external environment. This can be clearly seen: as the target approaches the asset, the algorithm dynamically shifts its resource allocation from the initially engaged radars (e.g., R7 and R8 at $t_{k}\leq 6$) to intermediate radars (e.g., R3 and R4, and notably R5 and R6 among the dominant radars at $t_{k}=15∼25$), and eventually to the radars closest to the asset (e.g., R1 and R2 at $t_{k}=36∼37$). Consequently, Figure 7c indicates that the compared methods exhibit a broadly comparable risk error range, which is strictly consistent with the equal-accuracy comparison setting. Therefore, the lower product values in Figure 7d strongly suggest that the proposed method achieves better resource efficiency without sacrificing overall operational risk estimation performance. The results suggest that the KNN algorithms suffer from severe resource inefficiency spikes when the geometric topology worsens (e.g., k=5 around $t_{k}=6$ and 12, or k=2 around $t_{k}=28$), whereas the proposed algorithm maintains the lowest product value throughout the entire time frame.

The superiority of the algorithm’s resource efficiency is more evident in Scenario 2, where the radars are geographically distributed. In this scenario, the proposed algorithm maintains a comparable risk error level while consuming less time than the KNN baselines over most time steps, with moderate peaks (e.g., around $t_{k}=19$ and 39) when the target enters regions with poor geometric topology. The heatmap in Figure 8b shows that the proposed method adaptively shifts the tracking burden from the initially closer radars (e.g., R1 and R2 at $t_{k}\leq 6$) to R5 and R6, and eventually to R7 and R8 at later time steps, while also using intermediate radars such as R3 and R4 to improve the viewing geometry. This behavior reflects a key advantage of the SOCP formulation: rather than simply favoring the nearest radars, it minimizes the maximum eigenvalue of the error covariance matrix, so a farther radar with a complementary line-of-sight can be selected over a closer radar with a redundant viewing angle. As a result, the proposed method avoids the severe risk error spikes often observed in distance-based heuristics under poor topologies, as supported by Figure 8c, and achieves the lowest or near-lowest time-error product in most frames in Figure 8d. The results in these two different scenarios indicate that the proposed algorithm adapts effectively to changes in the external environment.

It is worth noting that while the simulations focus on two distinct extreme phases—a target approaching the asset (Scenario 1) and a target moving away from it (Scenario 2)—these scenarios suggest the proposed algorithm’s adaptability to dynamic target trajectories. A passing-by scenario can be viewed as a sequential combination of approaching and receding phases, and the dynamic hand-over of radar resources observed in the heatmaps (e.g., Figure 8b) indicates that the method would maintain comparable performance under such conditions. These results demonstrate the algorithm’s robustness across diverse geometric topologies.

## 6. Conclusions

Previous research on cognitive radar mainly aimed to improve the accuracy of the target position. In this paper, an operational risk-aware approach is utilized in a safety-critical target tracking scenario with cognitive radar to allocate tracking resources by calculating the target’s operational risk accuracy. The operational risk is modeled as a monotonic function of the asset–target distance, with detailed parameters adjustable by the radar operator according to the operational environment. In particular, it is noteworthy that the proposed method can bring operational considerations into the decision process of cognitive radar. This approach can better reflect operational decision factors than previous methods, and this research can be extended by considering artificial intelligence techniques such as deep learning to train the operational decision-making process. In summary, the proposed operational risk-based SOCP framework does not primarily aim to outperform heuristic baselines by a large margin in raw risk error. Rather, its key advantage is that it achieves comparable operational risk estimation performance with substantially improved time-resource efficiency.

To establish the fundamental mechanism and mathematical validity of the operational risk-based resource allocation via SOCP, this study focused on a single-target scenario with an intuitive, distance-based operational risk function. However, the proposed framework lays the groundwork for more complex operational environments. In this paper, the proposed method was validated under a single-target assumption. However, practical radar network applications often involve multi-target tracking and severe resource competition among multiple targets. In future work, we plan to address these limitations by extending the algorithm to handle multi-target resource competition in dense clutter environments, integrating it with advanced data association techniques such as the Probabilistic Data Association Filter (PDAF). Furthermore, building upon the artificial intelligence techniques mentioned above, we aim to incorporate more sophisticated, data-driven operational risk modeling—such as AI-based composite operational risk functions—to evaluate diverse operational risks beyond the geometric distance.

## Figures and Tables

**Table 1:** Comparison of recent resource management approaches in radar networks.

Ref. and Method	Optimization Tool	Objective	Operational Risk
Liu et al. [13]	SOCP	Tracking Accuracy (CRLB)	No
Yan et al. [14]	Heuristics/Relaxation	Multitarget Tracking	No
Zhu et al. [20]	Reinforcement Learning	Target Localization	No
Tuncer et al. [6]	Constrained Opt.	Air Defense Tracking	Yes
Sun et al. [7]	Gaussian Processes	Maneuvering Tracking	No
**Proposed**	**SOCP**	**Risk Accuracy (Error)**	**Yes**

**Table 2:** System parameters used in the simulation.

Parameter	Value/Model
Number of Radars (*M*)	10
Target Motion Model	Constant Velocity (CV)
Sampling Interval ($T_{s}$)	10 s
Total Resource Budget ($T_{max}$)	25 ms
Reference SNR	10 dB
Effective Bandwidth (β)	0.33 MHz
Beamwidth ($\theta_{3dB}$)	10 mrad
Target RCS Model	Constant ($1\;m^{2}$)
Probability of Detection ($P_{d}$)	1 (Ideal)
False Alarm Rate ($P_{fa}$)	0 (Clutter-free)
Evaluation Metric	Risk Error

## Data Availability

Data available on request due to restrictions (e.g., privacy or ethical). The data presented in this study are available on request from the corresponding author.

## Appendix A. Derivation of the Positional PC-CRLB Eigenvalue Surrogate

Derivation: The eigenvalues of the positional covariance surrogate determine the principal axes of the corresponding error ellipse. In particular, the squared semi-major axis length is proportional to the maximum eigenvalue [13].

Let $p_{k}^{-}={\left[x_{k}^{-}\;y_{k}^{-}\right]}^{T}$ denote the positional subvector of the predicted target state. Using the block matrix structure of the Predicted Conditional Bayesian Information Matrix (PC-BIM) $J_{P}^{↑}\left(X_{k}^{-}\right)=\left[\begin{array}{cc} A & B\\ B^{T} & C \end{array}\right]$ [40], the positional PC-BIM is derived via the Schur complement as

(A1) $$J_{B}^{↑}\left(p_{k}^{-}\right)=J_{0,k}+\sum_{i=1}^{N}T_{i,k}^{d}J_{i,k},$$

where $J_{0,k}=A-BC^{-1}B^{T}$ is the prior information matrix, and $J_{i,k}=E_{X_{k}^{-}}\left[J\left(\varphi_{i,k}\right)\right]$ represents the information contribution from the *i*-th radar for i=1,…,N.

Since each $J_{i,k}$ is a symmetric positive semidefinite matrix and $J_{0,k}$ is symmetric, their positively weighted sum $J_{B}^{↑}\left(p_{k}^{-}\right)$ is a 2×2 symmetric matrix. Hence, it admits the eigendecomposition:

(A2) $$J_{B}^{↑}\left(p_{k}^{-}\right)=U_{k}\left[\begin{array}{cc} \mu_{1,k} & 0\\ 0 & \mu_{2,k} \end{array}\right]U_{k}^{T},$$

where $U_{k}$ is the orthogonal rotation matrix, and $\mu_{1,k},\mu_{2,k}>0$ are the eigenvalues under nonsingular geometry.

Accordingly, the equivalent positional PC-CRLB matrix is the inverse of the information matrix:

(A3) $$B_{p,k}^{-↑}={\left[J_{B}^{↑}\left(p_{k}^{-}\right)\right]}^{-1}=U_{k}\left[\begin{array}{cc} 1/\mu_{1,k} & 0\\ 0 & 1/\mu_{2,k} \end{array}\right]U_{k}^{T}.$$

This strictly implies that the eigenvalues of the covariance surrogate are the reciprocals of the information matrix’s eigenvalues:

(A4) $$\mathrm{eig}\;\left(B_{p,k}^{-↑}\right)=\left\{\frac{1}{\mu_{1,k}},\;\frac{1}{\mu_{2,k}}\right\}.$$

Therefore, the maximum eigenvalue of $B_{p,k}^{-↑}$ serves as a scalar surrogate for the dominant positional uncertainty.
